# CD40 Is Essential in the Upregulation of TRAF Proteins and NF-KappaB-Dependent Proinflammatory Gene Expression after Arterial Injury

**DOI:** 10.1371/journal.pone.0023239

**Published:** 2011-08-18

**Authors:** Zifang Song, Rong Jin, Shiyong Yu, Joshua J. Rivet, Susan S. Smyth, Anil Nanda, D. Neil Granger, Guohong Li

**Affiliations:** 1 Vascular Biology and Stroke Research Laboratory, Department of Neurosurgery, LSU Health Science Center in Shreveport, Shreveport, Louisiana, United States of America; 2 Department of Physiology, LSU Health Science Center in Shreveport, Shreveport, Louisiana, United States of America; 3 Department of Medicine, University of Kentucky, Lexington, Kentucky, United States of America; Universität Würzburg, Germany

## Abstract

Despite extensive investigations, restenosis, which is characterized primarily by neointima formation, remains an unsolved clinical problem after vascular interventions. A recent study has shown that CD40 signaling through TNF receptor associated factor 6 (TRAF6) plays a key role in neointima formation after carotid artery injury; however, underlying mechanisms are not clearly elucidated. Because neointima formation may vary significantly depending on the type of injury, we first assessed the effect of CD40 deficiency on neointima formation in 2 injury models, carotid artery ligation and femoral artery denudation injury. Compared with wild-type mice, CD40 deficiency significantly reduced neointima formation and lumen stenosis in two different models. Further, we investigated the mechanism by which CD40 signaling affects neointima formation after arterial injury. In wild-type mice, the expression levels of CD40, several TRAF proteins, including TRAF1, TRAF2, TRAF3, TRAF5, and TRAF6, as well as total NF-kB p65 and phospho-NF-kB p65, in the carotid artery were markedly upregulated within 3–7 days after carotid ligation. Deficiency of CD40 abolished the injury-induced upregulation of TRAFs including TRAF6 and NF-kB-p65 in the injured vessel wall. Further, CD40^−/−^ mice showed a significant decrease in the recruitment of neutrophils (at 3, 7d) and macrophages (at 7, 21d) into injured artery; this effect was most likely attributed to inhibition of NF-kB activation and marked downregulation of NF-kB-related gene expression, including cytokines (TNFα, IL-1β, IL-6), chemokines (MCP-1), and adhesion molecules (ICAM-1, VCAM-1). Moreover, neutrophil recruitment in a model of thioglycollate-induced peritonitis is impaired in CD40-deficient mice. In vitro data revealed that CD40 deficiency blocked CD40L-induced NF-kB p65 nuclear translocation in leukocytes. Altogether, our data identified for the first time that CD40 is essential in the upregulation of TRAF6, NF-kB activation, and NF-kB-dependent proinflammatory genes in vivo. Our findings firmly established the role for CD40 in neointima formation in 2 distinct injury models.

## Introduction

The CD40/CD40L system has been implicated in several cardiovascular diseases, such as atherosclerosis, ischemic heart disease, and stroke [1], [2]. Accumulating evidence indicates that CD40 signaling is mediated principally through tumor necrosis factor receptor–associated factors (TRAFs) [3], [4]. TRAFs are cytoplasmic adaptor proteins for the TNF/interleukin-1/Toll-like receptor superfamily. Ligands of this family comprise multiple pro-inflammatory cytokines such as CD40L, TNF-α, and interleukin-1β. TRAFs serve as adapter molecules linking CD40 to downstream signaling pathways. Different TRAFs exhibit specific biological functions [5]–[7]. The roles of individual TRAFs in the activation of different CD40-dependent signaling pathways have not been fully defined [7]. Establishing the significance and interplay of these roles will lead to a more complete understanding of mechanisms important to the CD40-mediated activation of the immune system and will reveal novel targets for the development of therapeutic agents [7].

The cytoplasmic domain of CD40 contains 2 independent membrane TRAF-binding domains: a proximal region binding TRAF6, and a distinct distal domain that binds TRAF-1/2/3/5 [4]. CD40-TRAFs signal transduction cascades are multiple and complex. Different TRAFs binding to CD40 triggers distinct signaling pathways leading to a variety of functional outcomes, depending on the cell type(s) involved [8]. For example, in monocytes and macrophages, TRAF6 is an essential mediator of CD40-activated proinflammatory pathways via activation of NF-κB and Src/ERK1/2 pathways [9], while in endothelial cells (ECs), inflammation is predominantly mediated via CD40-TRAF2 interactions [10]. However, there are currently no data available regarding whether such a CD40-mediated proinflammatory mechanism demonstrated in vitro also operates in vivo in response to vascular injury.

Although a pivotal role of CD40L in atherogenesis has been proven extensively [1], [3], [11], [12], only a few reports have investigated CD40-TRAF-dependent functions in the context of vascular injury. Miyahara et al [13] reported that among the TRAFs members, TRAF6 is predominantly involved in the process of inflammatory response and in-stent lesion formation in a stent implantation model of rabbit carotid artery. More recently, Donners et al [14] reported that the CD40-TRAF6 in bone marrow-derived cells plays a dominant role in neointima formation and arterial remodeling in a mouse carotid artery ligation model. However, there is clear evidence that the contribution of bone marrow-derived cells to neointima formation is rather limited in the mouse carotid artery ligation model.

In the present study, we investigated the role of CD40 in regulation of its adaptor proteins TRAFs and NF-kB-dependent proinflammatory genes in response to vascular injury. It is well known that neointima formation may vary significantly depending on the type of injury [14], [15]. Moreover, deficiency of the same genes (e.g. CD40L, integrin beta3) may have different and even opposite effects on neointima formation in different injury models [15], [16]. Thus, it is imperative to determine whether or not the CD40-mediated effect on neoinitma formation is model-specific. Therefore, we examined and compared the effect of CD40 deficiency on neointima formation and arterial remodeling using two distinct injury models, the guidewire-induced femoral artery denudation and the carotid artery ligation.

## Methods

### Ethics statement

All experimental procedures were carried out in accordance with the NIH Guide for the Care and Use of Laboratory Animals and approved by the Animal Care and Use Committee of the Louisiana State University Health Science Center-Shreveport (IACUC approval number: 0819). Male C57BL/6 mice and CD40^−/−^ mice (backcrossed onto a C57BL/6 background for >10 generations) were obtained from The Jackson Laboratory (Bar Harbor, ME).

### Two vascular injury models

In this study, two distinct types of mechanical vascular injuries were performed in the same mouse as previously reported [17]. Both left common carotid artery and left femoral artery of each mouse were injured to induce neointima formation. Mice were anesthetized with intraperitoneal administration of ketamine (80 mg/kg body wt; Abbott Laboratories) and xylazine (5 mg/kg body wt; Rompun, Bayer Corp). Carotid artery ligation was performed as described previously [18]. In brief, the left common carotid artery was exposed through a small midline incision in the neck and the artery was completely ligated with a 7-0 silk suture just proximal to the carotid bifurcation to disrupt blood flow. Endothelial denudation of mouse femoral arteries was performed as previously described [19] In brief, the left femoral artery and its muscular branch were exposed, and a 0.014′ (0.36 mm) diameter angioplasty guide wire was introduced into the arterial lumen and advanced to the level of the aortic bifurcation and pulled back three times. Removal of the endothelium was confirmed by *in vivo* Evan's blue staining in injured arteries compared with noninjured arteries (Figure S1). Animals were sacrificed at defined time points after injury. After in situ cardiac perfusion with 4% paraformaldehyde or saline, arteries were collected for histology or biochemical analysis as previously described [19], [20].

### Histology and morphometry

The arterial segments were dehydrated in ethanol and xylene and embedded in paraffin. Serial cross-sections (5 µm thick) were cut beginning 100 µm proximal to the carotid ligation site or the femoral bifurcation. Histomorphometric analysis was performed at 7 cross-section levels (with 120 µm intervals) (Figure S3A). For each level, three cross-sections were stained with Verhoeff's Elastic staining and morphometric analysis was performed by an individual blinded to experimental design. We measured the lumen area, intimal area (the area within the internal elastic lamina [IEL] minus the lumen area), medial area (defined as the area within the external elastic lamina [EEL] minus the area within the IEL), and total vessel area (the area encompassed by the EEL) at each level as previously described [14], [20]. The mean value of neointimal area, intima/media ratio, lumen stenosis ratio, and total vessel area was calculated over 7 cross-section levels.

### Immunohistochemistry

Paraffin-embedded sections were stained with the avidin-biotin-peroxidase method (Vector Laboratories). The following primary antibodies (from Santa Cruz, unless otherwise indicated) were used: CD40 (1∶1000), TRAF1 (1∶25), TRAF2 (1∶50), TRAF3 (1∶1000), TRAF5 (1∶400), TRAF6 (1∶50), ICAM-1 (1∶50), VCAM-1 (1∶50), MCP-1 (1∶1000), NF-kB p65 (1∶750; Abcam), Phospho-p65 (phosphor Ser536; 1∶50; Abcam), Mac-2 (1∶1000; M3/38, Accurate) to detect monocyte/macrophages, and anti-PMN mAb (1∶2500; Accurate) to detect neutrophils. Isotype-matched antibodies served as negative controls. Sections were incubated with the appropriate secondary antibody (Vector Laboratories) and visualized by 3,3′-diaminobenidine (DAB; Vector Laboratories) and counterstained with hematoxylin. For quantitative comparison of leukocyte infiltration, the percent of the positively stained cells to the total cells was calculated. For quantitative comparison of the expression of indicated molecules, the percent of the positively stained area to the total traced area was determined. Morphometric and immunohistochemical analysis were performed by one blinded investigator using Image Pro Plus 5.0 (Media Cybernetics).

### Real-time RT-PCR

Total RNA was extracted from pooled carotid arteries (n = 5) using TRIzol Reagent (Invitrogen) and treated with DNase I to remove genomic DNA. A quantitative real-time reverse-transcriptase (RT)-PCR was performed with a Bio-Rad thermocycler and an SYBR green kit (Bio-Rad) according to the recommendations of the manufacturer. Sequence-specific primers used for the reaction are presented *in Table S2*. The relative mRNA expression was normalized by GAPDH RNA levels. Data are expressed as the fold change compared to uninjured arteries.

### Thioglycollate-elicited peritonitis model

Peritoneal recruitment of leukocytes was induced using 2.0 ml of sterile 3% thioglycollate (Sigma-Aldrich) as we described previously [20]. After 5 hours, mice were killed, the peritoneal cavity was rinsed with 5 mL ice-cold PBS containing 0.1% BSA and 20 µmol/L disodium EDTA. Cell viability (trypan blue exclusion) was >95% and Cytospin preparations stained with Giemsa revealed that >90% of the cells were neutrophils. The number of neutrophils in the peritoneal fluid was counted with a hemocytometer (Hausser Scientific).

### NF-kB p65 nuclear translocation assay

Isolated neutrophils were resuspended in RPMI 1640 containing 0.5% (w/v) low-endotoxin BSA. The effect of CD40 on the nuclear translocation of *NF-kB* p65 was examined by immunocytochemical method as previously described [21], [22]. In brief, freshly isolated neutrophils were stimulated with 1.0 µg/ml CD40L (R&D Systems) for 1 h at 37°C. The cells were fixed with 4% PFA in PBS (pH 7.4) for 20 min at room temperature, followed by permeabilization with 0.2% Triton X-100. Non-specific binding was blocked with 5% normal goat serum (Invitrogen) for 1 h. Then cells were incubated with rabbit polyclonal anti-human p65 antibody (1∶100, Abcam) overnight at 4°C, followed by incubation with Alexa Fluor 488-conjugated goat anti-rabbit IgG (1∶200, Invitrogen) for 1 h at room temperature. Nuclei were stained with DAPI (Vector Laboratories). Images were taken using a fluorescence microscope (Nikon, Japan) and quantification was performed using Image-Pro Plus software. The number of total cells and nuclei-positively stained cells were counted from eight randomly chosen high-power (×200) fields in each well. Each assay was performed in quadruplicate.

### Statistical analysis

Data are presented as means ± SEM and were determined using either two-tailed t-test analysis or one-way ANOVA followed by Fisher's exact test analysis. *P* values less than 0.05 were considered statistically significant.

## Results

### CD40 deficiency inhibits up-regulation of TRAFs in response to vascular injury

**Figure 1:**
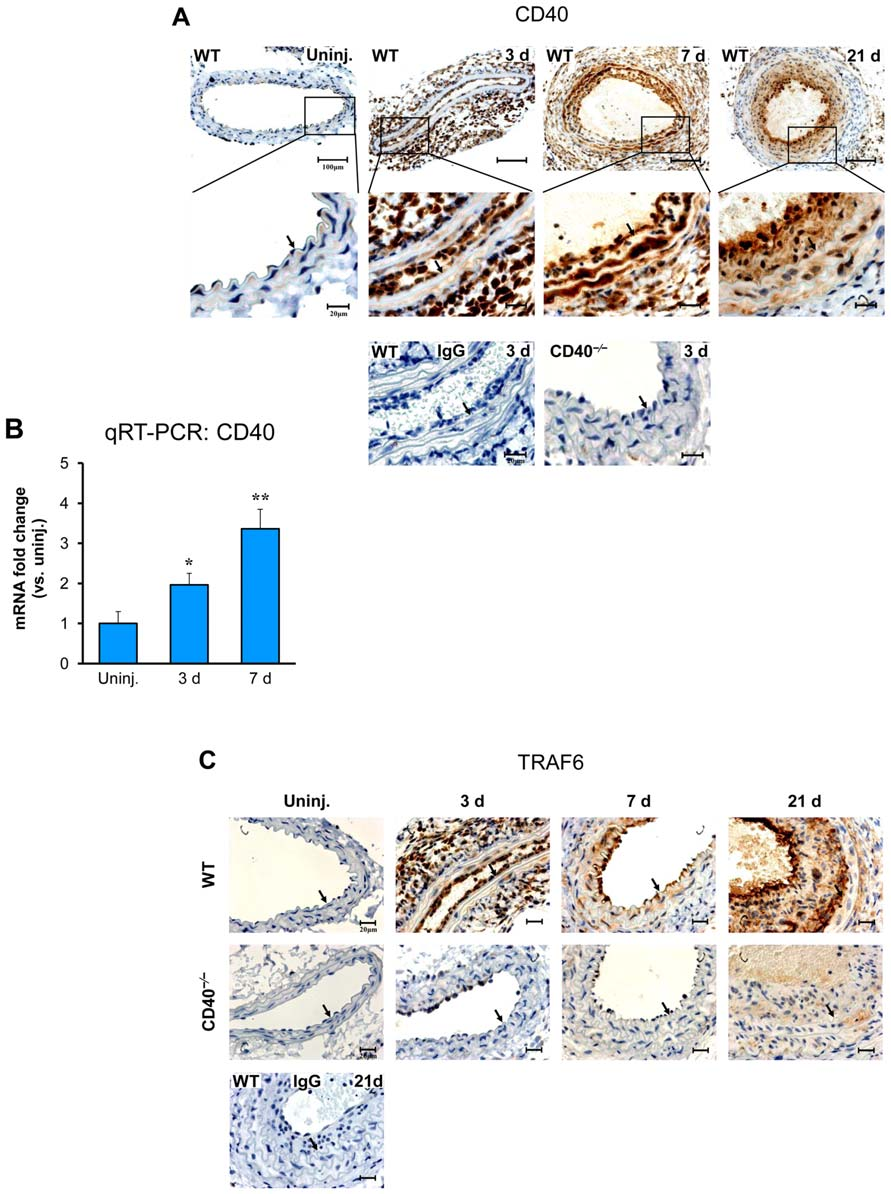
Expression of CD40 and TRAFs in the carotid artery wall after ligation injury. (**A**) Representative images of immunostaining for CD40 in carotid arteries from WT and CD40^−/−^ mice (n = 5 per group). Scale bars: 20 µm or 100 µm. (**B**) Quantitative RT-PCR analysis of CD40 expression in WT carotid arteries (n = 5 per group). mRNA levels are normalized to GAPDH. Data are expressed as mean ± SEM. * *P*<0.05 and ** *P*<0.01 versus uninjured (uninj.)control. (**C**) Representative images of immunostaining for TRAF6 in carotid arteries from WT and CD40^−/−^ mice (n = 5 per group). Scale bars: 20 µm. Arrows indicate the internal elastic lamina.

Overexpression of CD40 and its adaptor proteins TRAFs have been observed in established atherosclerotic plaques and in developed neointimal lesions (28d) after carotid artery ligation [14], [23]. Using immunohistochemistry, we examined the expression pattern and changes for CD40 and TRAFs in the arterial wall at different time points after carotid artery ligation. Our data showed a significant increase in CD40 expression both in the early phase (3d, 7d) and the late phase (21d) in WT mice (Figure 1A), and the increase in CD40 was accompanied by a parallel increase in TRAFs, including TRAF6 (Figure 1C) and TRAF1/2/3/5 (Figure S2). Increased expression of CD40 in the injured arterial wall was confirmed at the mRNA level as determined by real-time RT-PCR (Figure 1B). In contrast, the injury-induced expression of all TRAFs was almost completely abolished in the CD40-deficient mice at all time points after the injury. Our data showed for the first time that CD40 is essential for the upregulation of TRAF proteins in response to vascular injury.

### CD40 deficiency inhibits proinflammatory gene expression and leukocytes recruitment

Recruitment of circulating leukocytes to the injured arterial wall has been shown to be an initial step and play a critical role in vascular inflammatory response and neointima formation [24]. Previous data have shown that CD40 deficiency reduced the infiltration of inflammatory cells, including CD45+ total leukocytes and CD3+ T cells in the developed (28d) neointimal lesions [14], but the underlying mechanism remains largely unknown. In the present study, we examined by immunohistochemistry the effects of CD40 deficiency on leukocuyte recruitment at different time points and explored the molecular mechanisms involved.

**Figure 2:**
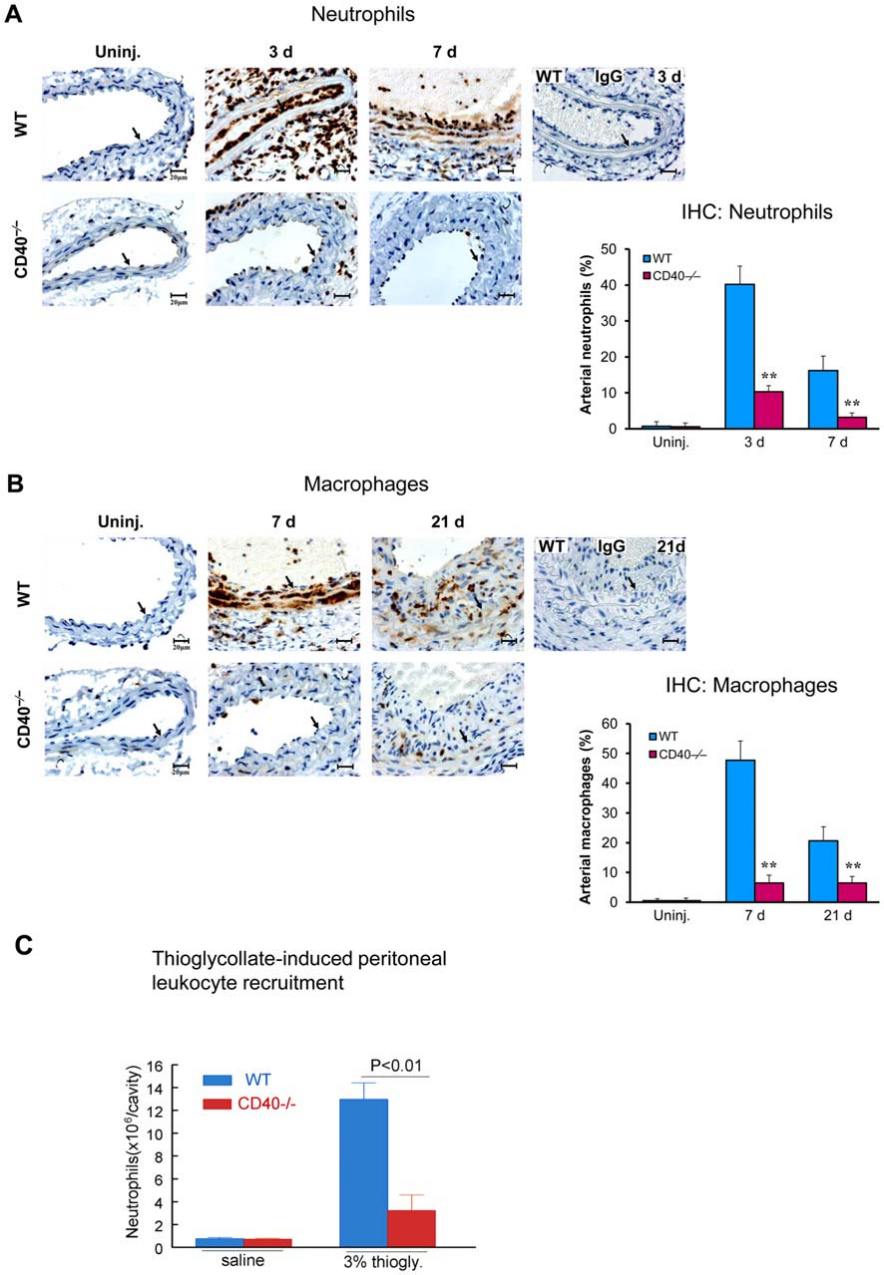
CD40 deficiency inhibits leukocytes recruitment into the carotid artery wall after ligation injury, and thioglycollate-induced peritoneal leukocyte recruitment. Representative images of immunostaining for neutrophils (anti-PMN mAb) (**A**) and for Mac-2-positive monocyte/macrophages (**B**) in carotid arteries from WT and CD40^−/−^ mice (n = 5 per group). Positively stained cells were quantified as described in *Method*. Arrows indicate the internal elastic lamina. Scale bars: 20 µm. Data are expressed as mean ± SEM. ** *P*<0.01 versus corresponding WT. (**C**) Neutrophil recruitment in a model of thioglycollate-induced peritonitis is impaired in CD40-deficient mice. Mice were treated i.p. with 2 ml of 3% thioglycollate or the same volume of isotonic saline. 5 hours later, total number of neutrophils (×10^6^) in the peritoneal cavity was counted. n = 5 mice/group.

In wild-type mice, neutrophil infiltration was dramatically increased at 3d after carotid injury, prominently seen in the adventia and on the lumen surface, but modest in the media (Figure 2). However, neutrophil infiltration became very evident in the media at 7 days after the injury (Figure 2A). Mac-2+monocyte/macrophages were limited at 3 days (data not shown), but became evident in the media (7d) and then in the developing neointima (21d) (Figure 2B). In CD40^−/−^ mice, absence of CD40 profoundly reduced the number of infiltrated neutrophils and monocyte/macrophages at all the indicated time points after carotid artery ligation (Figure 2). In addition, we observed that neutrophil recruitment in a model of thioglycollate-induced peritonitis is impaired in CD40-deficient mice (Figure 2C). The reduction in leukocytes recruitment into the injured vessel wall and the peritoneal cavity of CD40^−/−^ mice was not due to lower levels of blood leukocytes, because complete blood cell count results showed no detectable differences in levels of total leukocytes or specific leukocyte subtypes between CD40^−/−^ and WT mice (Table S1).

**Figure 3:**
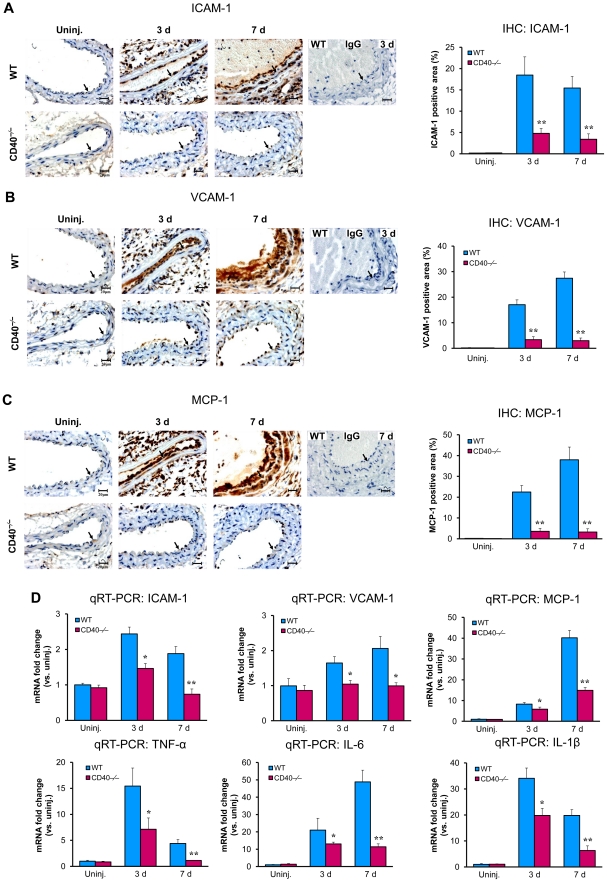
CD40 deficiency inhibits expression of proinflammatory mediators in the carotid artery wall after ligation injury. Representative cross-sections from carotid arteries immunostained for ICAM-1 (**A**), VCAM-1 (**B**), and MCP-1 (**C**), as well as their quantitative analysis, in WT and CD40^−/−^ mice (n = 5 per group). Arrows indicate the internal elastic lamina. Scale bars: 20 µm. Data are expressed as mean ± SEM. ** *P*<0.01 versus corresponding WT. (**D**) Quantitative RT-PCR analysis of mRNA expression of ICAM-1, VCAM-1, MCP-1, TNF-α, IL-6, and IL-1β, in WT and CD40^−/−^ carotid arteries from WT and CD40^−/−^ mice (n = 5 per group). mRNA levels are normalized to GAPDH. Data are expressed as mean ± SEM. * *P*<0.05 and ** *P*<0.01 versus corresponding WT.

To elucidate the molecular mechanisms underlying the CD40-mediated effects on leukocyte recruitment, we examined the expression of proinflammatory genes in the ligated carotid arteries. Immunohistochemical staining demonstrated that the expression of adhesion molecules (ICAM-1, VCAM-1) and chemokine MCP-1 was markedly increased at 3 and 7 days after injury in wildtype mice (Figure 3, A to C), but the increase of all these proinflammatory mediators was markedly blocked in CD40^−/−^ mice at all time points (Figure 3, A to C). Consistent with immunostaining results, real-time RT-PCR analysis confirmed a significant reduction in mRNA expression of ICAM-1, VCAM-1, and MCP-1 in the CD40-null carotid arteries compared with the WT arteries at 3 and 7 days after injury (Figure 3D). Moreover, real-time RT-PCR analysis demonstrated that the mRNA levels of proinflammatory cytokines including TNF-α, IL-6, and IL-1β, were markedly increased in the WT carotid arteries at 3 and 7 days after injury, but the levels were much lower in CD40^−/−^ mice (Figure 3D). These cytokines are known to potently stimulate expression of various adhesion molecules and chemokines in vascular cells and leukocytes in vivo and in vitro [25]. Plasma levels of MCP-1 and soluble VCAM-1 in CD40−/− mice were lower than WT controls, but not reaching statistical significance (Figure S5).

### CD40 deficiency inhibits NF-κB activation in the arterial wall in response to vascular injury and in leukocytes in vitro

**Figure 4:**
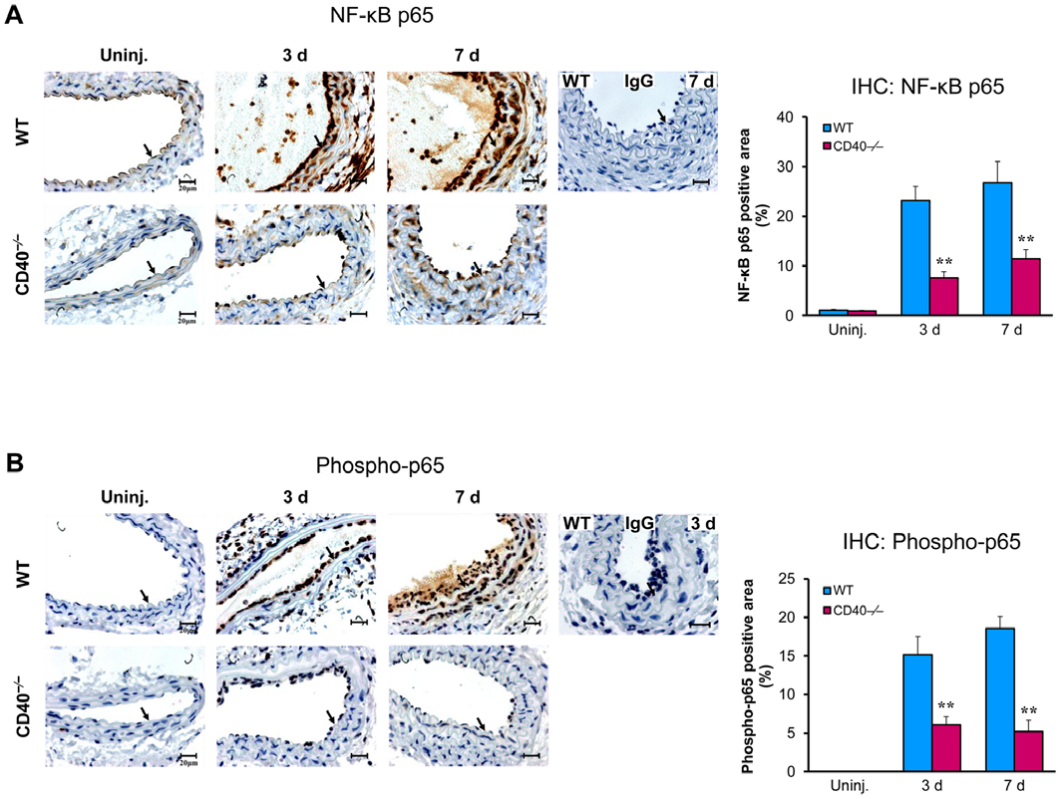
CD40 deficiency inhibits NF-κB activation in the carotid artery wall after ligation injury. Representative cross-sections from carotid artery immunostained for total NF-κB p65 (**A**) and activated phospho-p65 (Ser536) (**B**), as well as their quantitative analysis, in WT and CD40^−/−^ mice (n = 5 per group). Arrows indicate the internal elastic lamina. Scale bars: 20 µm. Data are expressed as mean ± SEM. ** *P*<0.01 versus corresponding WT.

In vitro data have shown that CD40-TRAF interaction triggers activation of NF-κB, a major transcription factor that regulates expression of many different proinflammatory genes [26], [27], including those genes we examined above. However, there is no in vivo data indicating whether/how CD40 mediates vascular inflammation through a NF-kB-dependent mechanism after vascular injury. We examined NF-kB activation by immunostaining of total NF-kB p65 and its activated form of phospho-p65 in the injured carotid artery. In wildtype mice, immunostaining for both p65 and phosphor-p65 was markedly increased at 3 and 7days after carotid ligation (Figure 4, A and B), findings in general agreement with previous studies [28]–[30]. In CD40^−/−^ mice, the injury-induced upregulation of p65 and phosphor-p65 was largely inhibited at both time points (Figure 4, A and B).

**Figure 5:**
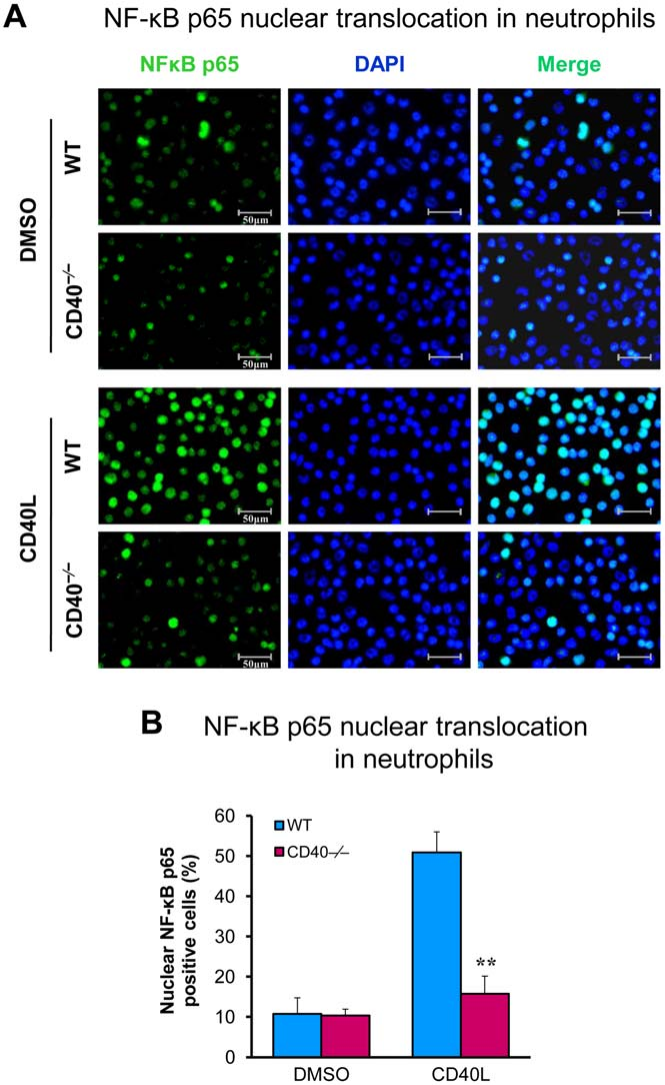
CD40 is required for CD40L-induced activation of NF-κB pathway in neutrophils. (**A**) NF-κB nuclear translocation was assessed by immunofluorescence staining for NF-κB p65 (green) in wildtype and CD40-deficient neutrophils exposed to CD40L (1.0 µg/ml) for 1 h. Cell nuclei were detected by DAPI (blue). Scale bars: 50 µm. (**B**) NF-κB nuclear translocation was assessed and quantified as the percentage of the p65 nuclei-positively stained cells to the total cells. Similar results are obtained from three independent experiments. Data are expressed as mean ± SEM. ** *P*<0.01 versus WT.

Because previous data show that leukocyte CD40 plays a predominant role in neointima formation after carotid artery ligation [14], we further examined whether CD40 is required for CD40L-induced activation of NF-κB pathway in leukocytes. Translocation of cytoplasmic NF-κB p65 to the nucleus is a key step in activation of the NF-κB pathway [26], [27]. By immunofluorescence staining, we demonstrated that CD40L stimulation induced NF-kB p65 translocation from the cytoplasm to the nucleus in wild-type neutrophils, but this effect was largely inhibited in CD40-deficient neutrophils (Figure 5).

Taken together, our data provide the first direct in vivo evidence that CD40 plays a critical role in mediating proinflammatory gene expression and leukocyte infiltration possibly via activation of NF-kB-dependent mechanism.

### CD40 deficiency reduces neointima formation in two different injury models

Because neointima formation may vary significantly depending on the nature of vascular injury, we examined and compared the effects of CD40 deficiency on neointima formation and arterial remodeling using two different injury models. In the carotid artery ligation model, our results reproduced previous observations [14]. Compared with wildtype mice, neointimal size and intima/media ratios were significantly reduced in CD40^−/−^ mice, with significant reduction in lumen stenosis (Figure S3). Consistent with the published data [14], we showed that CD40^−/−^ mice exhibited impaired arterial remodeling after carotid ligation, i.e. total vessel size of CD40^−/−^ mice was significantly smaller than that of wild-type mice (Figure S4A). It could not be explained by differences in carotid geometry, since there were no differences in uninjured carotid arteries between both genotypes, with respect to the lumen area, media area, and total vessel area (Figure S4B).

**Figure 6:**
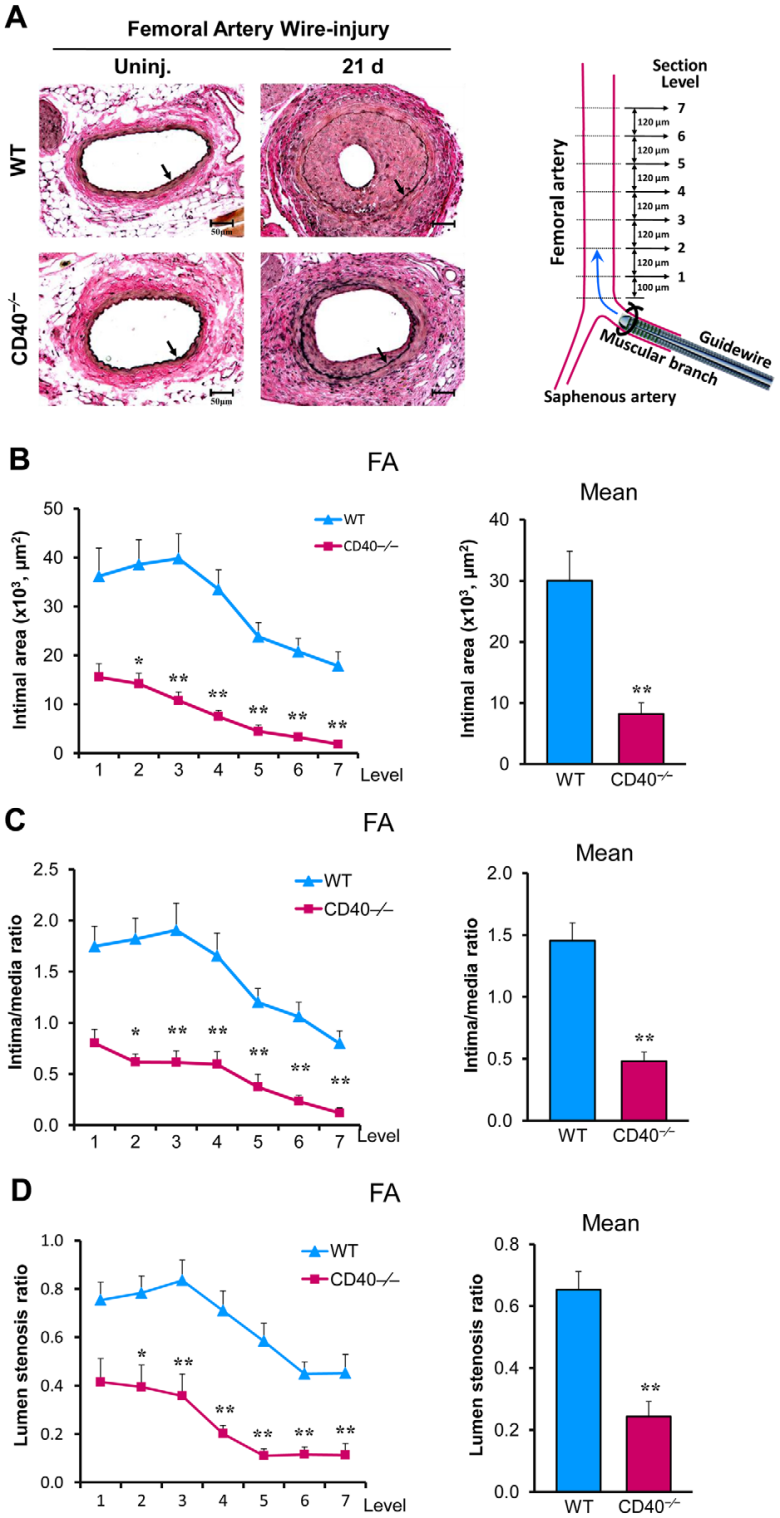
Decreased neointima formation and lumen stenosis after femoral artery wire-denudation in CD40^−/−^ mice. (**A**) Representative Elastic-stained sections (level 3) of femoral arteries 21d after injury in WT and CD40^−/−^ mice (left panel). Schematic diagram of femoral artery guidewire-induced injury and tissue microtomy (right panel). Arrows indicate the internal elastic lamina. Scale bars: 50 µm. (**B**) Intima was measured at the 7 cross-section levels (120-µm intervals), and their mean intimal area was calculated. Intima/media ratio (**C**) and lumen stenosis ratio (**D**) at each level as well as their mean ratios were determined. n = 10 mice per group. Data are expressed as mean ± SEM. * *P*<0.05 and ** *P*<0.01 versus corresponding WT.

Wire denudation injury can induce significant neointima formation in the femoral artery, although the extent of neointimal lesion often varies among different laboratories. The extent of neointima formation of WT mice on C57BL/6 background shown in our study was similar to those reported by other laboratories [31], [32]. In this model, we demonstrated that neointimal size, intima/media ratios, and lumen stenosis were significantly reduced in CD40−/− mice (Figure 6), findings in agreement with those obtained in the carotid ligation model. It warrants to note that, unlike carotid artery ligation model, vascular remodeling was not impaired in CD40^−/−^ mice compared with wild-type mice after femoral artery denudation, i.e. total vessel size was similar in both types of mice (Figure S4A). In addition, no difference in vessel geometry were found in uninjured femoral arteries between both genotypes (Figure S4C).

## Discussion

This study elucidates a cellular/molecular mechanism of the CD40 signaling involved in the response to vascular injury: increased expression of CD40 and its adaptor TRAF proteins and activation of the NF-kB-dependent proinflammatory pathway. We observed that CD40 deficiency profoundly inhibits expression of TRAF proteins and activation of NF-kB that involves gene expression of proinflammatory cytokines and adhesion molecules in the injured arterial wall and thus, the CD40 signaling pathway plays a crucial role in mediating inflammatory response and leukocyte recruitment in vivo. The results also firmly establish the functional role of CD40 in neointima formation in two distinct vascular injury models.

Overexpression of CD40 and TRAFs (-1,-2,-3,-5,-6) has been observed in murine and human atherosclerotic lesions [23], [33]. There are contridicatory reports about the role of CD40 in atherogenesis. Although overall deletion of CD40 did not limit atherogenesis in LDLr^−/−^CD40^−/−^ mice [34], overall deletion of CD40 significantly limited atherogenesis in apoE^−/−^CD40^−/−^ mice [35]. The exact reasons for this discrepancy are unclear at present. Increased expression of CD40 and several TRAFs including TRAF6 has also been observed in developed neointimal lesions after vascular injury [14]. In vitro data show that CD40L and other proinflammatory cytokines modulate TRAFs differentially in ECs, SMCs, and macrophages [23]. For example, ligation of CD40 by CD40L enhanced the expression of TRAF-1, -2, -3, and 6, but not TRAF-5 in ECs. Stimulation with IL-1β or TNF-α induced expression of TRAF-1, -3, and -6 in ECs and SMCs, whereas neither stimulus affected the expression of TRAF-2 or -5 [23]. However, it remains unknown about how CD40 and TRAFs are upregulated in the damaged arterial wall in vivo. In the present study, we used CD40-deficient mice to determine whether CD40 is required for the injury-induced expression of TRAFs at different time points after carotid artery ligation. We observed that absence of CD40 almost completely abolished the induction of TRAFs, indicating an essential role of CD40 in the upregulation of TRAF proteins after vascular injury.

It has been reported that different TRAFs differentially contribute to the development of atherosclerosis. Lutgens et al [32] reported that deficient CD40-TRAF6 signaling in leukocytes prevents atherosclerosis in apolipoprotein E knockout mice. In contrast, Stachon et al [36] reported that TRAF6 deficiency does not influence atherogenesis in LDLR−/− mice. Missiou et al [37], [38] reported that TRAF5 deficiency accelerates, by contrast, TRAF1 deficiency attenuates atherogenesis in LDLR−/− mice. More recently, Donners et al [14] demonstrated that bone marrow-derived leukocyte CD40 appeared to play a dominant role in neointima formation after carotid artery ligation, but the role of CD40 expressed by non-hematopoietic cells (vascular wall cells) was not examined in the study.

Emerging evidence indicates that many of the biological effects of TRAF signaling appear to be mediated through the activation of transcription factor NF-κB [7], [39]. NF-κB plays a key role in regulating the expression of many cytokines and adhesion molecules involved in atherosclerosis and neointima formation after vascular injury. The activation of NF-κB in the vessel wall after arterial injury in rats and mice has been established by several reports and has been correlated to the induced expression of NF-κB-dependent proinflammatory genes such as VCAM-1 and MCP-1 [40]–[42]. Inhibition of NF-κB activity in injured arteries by overexpression of IκBα [41] blockade of IKKβ [42] or with NF-κB decoy [43] inhibited vascular inflammatory responses and neointimal formation. To best of our knowledge, however, no in vivo studies have documented the role of CD40 in regulation of NF-kB activation and NF-kB-dependent gene expression in the injured/diseased arteries. The present study observed robust activation of NF-κB in the carotid artery wall in the early phase (3, 7d) after injury, accompanied by increased expression of cytokines (TNF-α, IL-1β, IL-6), chemokine MCP-1, and adhesion molecules (ICAM-1, VCAM-1), as well as abundant recruitment of neutrophils sand monocyte/macrophages. In vitro, the present data confirmed that CD40 deficiency inhibited CD40L-induced NF-kB nuclear translocation in neutrophils. Notably, the activation of NF-kB and the increased expression of all these genes and leukocyte recruitment all are markedly inhibited in CD40−/− mice.

Proinflammatory cytokines, TNF-α, IL-1β, and IL-6, play a key role in mediating adhesion molecule expression and immune cell activation [24], [44]. ICAM-1 and VCAM-1 are the adhesion molecules of immunoglobulin family responsible for the firm arrest and transmigration of leukocytes into inflammatory sites [45]. MCP-1 is a key chemokine that stimulate monocyte adhesion and accumulation in diseased vessel [44]. All these proinflammatory mediators have been implicated in the process of neointima formation after vascular injury [24]. Inhibition of cytokines or adhesion molecules decreased neointima formation after vascular injury [46], [47]. Therefore, we conclude that CD40 deficiency reduces leukocyte recruitment and neointima formation through downregulating activation of NF-κB and NF-kB-dependent upregulation of multiple pro-inflammatory genes in the injured arterial wall.

The carotid artery ligation model and the femoral artery wire denudation model are both widely used to investigate the pathophysiology of neointima formation after vascualr injury. In the ligation model, the arterial endothelium and the internal elastic lamina (IEL) and the external elastic lamina (EEL) are generally intact (Figs. S1B, S3A). In the wire injury model, the arterial endothelium are removed (Fig. S1A), the medial layer including IEL is partially damaged, but EEL is generally intact (Figure 6A). The compositions of the neointimal lesions in both models include SMC-like cells, infiltrating leukocytes and extracellualr matrix. But, the exact cellular composition of the lesions may be different significantly among models [17], [18], [48]. It is well known that neointima formation might be diverse depending on the type of vascular injury [16], [17]. For example, genetic deletion of the integrin β3 significantly reduced neointima formation after carotid artery ligation, but increased neointima formation after femoral artery wire-denudation injury [16]. Similarly, genetic deletion of CD40L significantly increased neointima formation after carotid collar injury [15], but decreased neointima formation after carotid artery ligation [14]. Therefore, it is particularly important to establish the role of CD40 in neointima formation using different vascular injury models. Increasing evidence has shown that the CD40L-mediated effects are not necessarily identical to the CD40-mediated effects [8]. This is because that CD40 is not the only receptor for CD40L, e.g. CD40L can bind to other receptors such as GPIIb/IIIa in platelets and Mac-1 in leukocytes [8]. Likewise, CD40L is not the only protein that interacts with CD40, e.g. other proteins such as HSP70 can interact with CD40 [49]. The present study firmly established the role of CD40 deficiency in neointima formation using carotid artery ligation model and guidewire-induced femoral artery denudation model performed in the same mouse. Neointimal lesions and lumen stenosis were consistently reduced in CD40^−/−^ mice in both models. Only difference is that arterial remodeling was impaired in the ligated carotid artery, but not impaired in the wire-denudated femoral artery of CD40−/− mice. The mechanism is not clear at present.

Limitations of the study and future research: Several important questions remain to be addressed in the future research. For example, what are the cell types in the 2 different injury models that express CD40 and the respective TRAF molecules? Which NF-kB pathway is activated/prevented, canonical or non-canonical, and which TRAFs are involved in the canonical/non-canonical pathway in neointima formation?

In summary, the novelty of the present study is that we identified for the first time that CD40 is essential in the upregulation of TRAF proteins and NF-kB-dependent proinflammatory genes in vivo. Also importantly, our study firmly established the role for CD40 in neointima formation in 2 distinct vascular injury models. These findings may provide valuable information for the development of new therapeutic interventions against vascular restenosis.

## Supporting Information

Figure S1 **Removal of the endothelium was confirmed by in vivo Evan's blue staining 4 h after vascular injury.** Briefly, 1% Evans blue (in 200 ìL saline) was injected intracardially 10 minutes before sacrifice, followed by perfusion fixation with 10% formalin for 5 minutes. Arteries were opened longitudinally and placed en face between microscopic slides. A, Wire-injured left and uninjured right femoral artery (FA). B, (left) Wire-injured common carotid artery (CCA), and (right) suture ligation-injured CCA.
(PDF)

Figure S2 **Effects of CD40 deficiency on TRAFs expression in the carotid artery wall after ligation injury.** Representative cross-sections from carotid arteries immunostained for TRAF1 (**A**), TRAF2 (**B**), TRAF3 (**C**), TRAF5 (**D**) in WT and CD40^−/−^ mice (n = 5 per group). Arrows indicate the internal elastic lamina. Scale bars: 20 µm.
(PDF)

Figure S3 **Decreased neointima formation and lumen stenosis after carotid artery ligation in CD40^−/−^ mice.** (**A**) Representative Elastic-stained sections (level 3) of carotid arteries 21d after injury in WT and CD40^−/−^ mice (left panel). Schematic diagram of carotid artery ligation and tissue microtomy (right panel). Arrows indicate the internal elastic lamina. Scale bars: 50 µm. (**B**) Intima was measured at the 7 cross-section levels (120-µm intervals), and their mean intimal area was calculated. Intima/media ratio (**C**) and lumen stenosis ratio (**D**) at each level as well as their mean ratios were determined. n = 10 mice per group. Data are expressed as mean ± SEM. * *P*<0.05 and ** *P*<0.01 versus corresponding WT.
(PDF)

Figure S4 **Effects of CD40 deficiency on arterial remodeling after injury.** (**A**) Total vessel area was measured in carotid arteries and femoral arteries 21d after injury in WT and CD40^−/−^ mice. n = 10 mice per group. Data are expressed as mean ± SEM. ** *P*<0.01 versus WT. Lumen area, media area, and total vessel area were measured in uninjured carotid arteries (**B**) and uninjured femoral arteries (**C**) from WT (n = 7 vessel) and CD40^−/−^ mice (n = 6 vessel). Data are expressed as mean ± SEM. No differences in vessel geometry were found between WT and CD40^−/−^ mice in uninjured arteries.
(PDF)

Figure S5 **Measurement of plasma levels of MCP-1 and soluble VCAM-1 using Quantikine Mouse ELISA Kits (R&D Systems) in CD40−/− mice and WT controls.** NS: P>0.05. n = 5/group.
(PDF)

Table S1 **Complete blood cell counts.**
(PDF)

Table S2 **Primer sequences for real-time RT-PCR.**
(PDF)
