# Supplementary material for: CD40 Is Essential in the Upregulation of TRAF Proteins and NF-KappaB-Dependent Proinflammatory Gene Expression after Arterial Injury
Source: PLoS One. 2011 Aug 18;6(8):e23239. doi: 10.1371/journal.pone.0023239 (PMC3158063; doi:10.1371/journal.pone.0023239)
Supplement: Figure S1 — Removal of the endothelium was confirmed by in vivo Evan's blue staining 4 h after vascular injury. Briefly, 1% Evans blue (in 200 ìL saline) was injected intracardially 10 minutes before sacrifice, followed by perfusion fixation with 10% formalin for 5 minutes. Arteries were opened longitudinally and placed en face between microscopic slides. A, Wire-injured left and uninjured right femoral artery (FA). B, (left) Wire-injured common carotid artery (CCA), and (right) suture ligation-injured CCA. (PDF) [file pone.0023239.s001.pdf]

Figure S1. **Endothelial removal was confirmed by in vivo Evans blue staining**

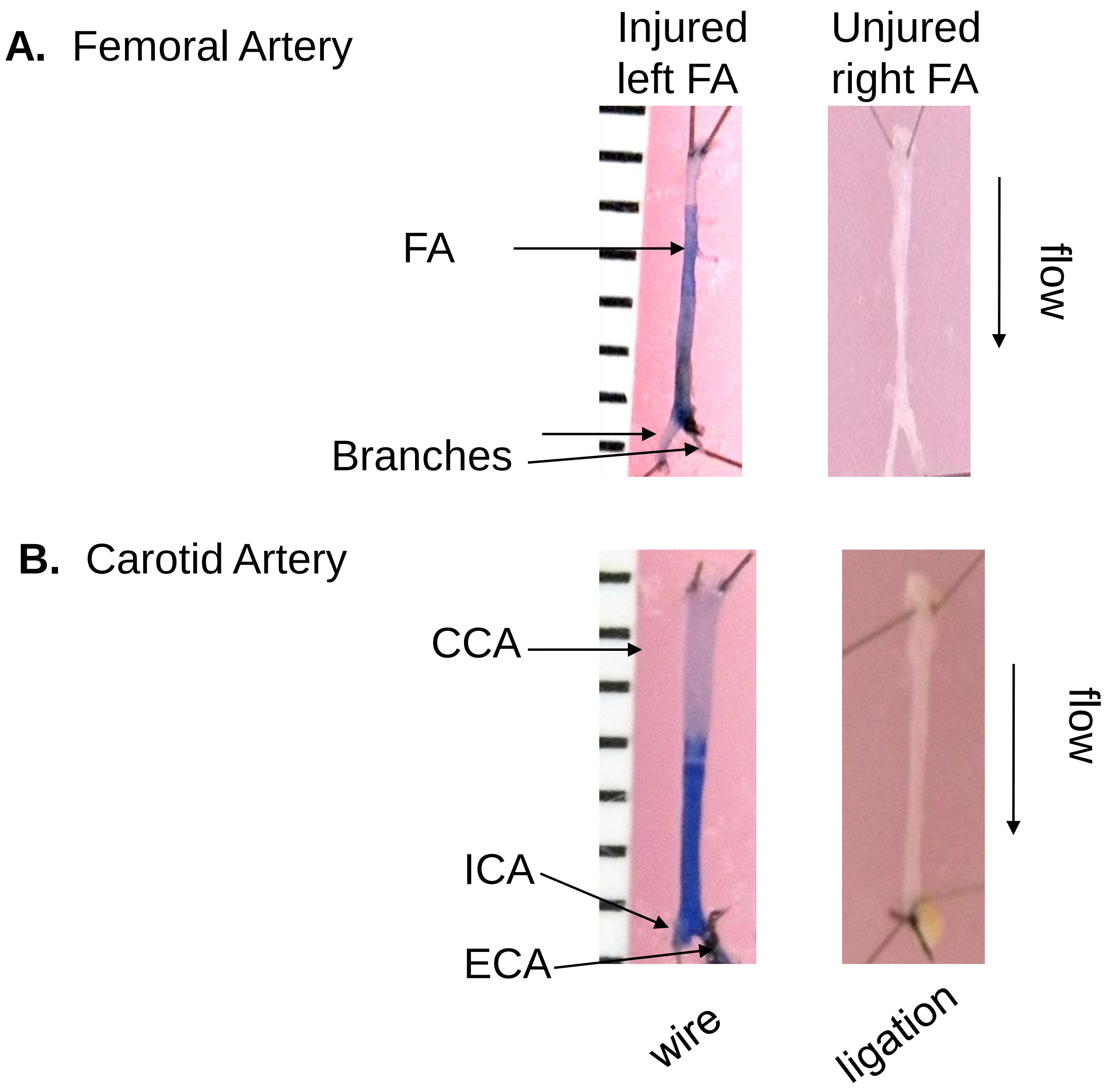
A. Femoral Artery
Injured left FA
Unjured right FA
FA
Branches
flow
B. Carotid Artery
CCA
ICA
ECA
flow
wire
ligation

Fig. S1.
