# Supplementary material for: CD40 Is Essential in the Upregulation of TRAF Proteins and NF-KappaB-Dependent Proinflammatory Gene Expression after Arterial Injury
Source: PLoS One. 2011 Aug 18;6(8):e23239. doi: 10.1371/journal.pone.0023239 (PMC3158063; doi:10.1371/journal.pone.0023239)
Supplement: Figure S2 — Effects of CD40 deficiency on TRAFs expression in the carotid artery wall after ligation injury. Representative cross-sections from carotid arteries immunostained for TRAF1 (A), TRAF2 (B), TRAF3 (C), TRAF5 (D) in WT and CD40−/− mice (n = 5 per group). Arrows indicate the internal elastic lamina. Scale bars: 20 µm. (PDF) [file pone.0023239.s002.pdf]

Figure S2. **CD40 deficiency inhibits TRAFs expression in the vessel wall after carotid ligation**

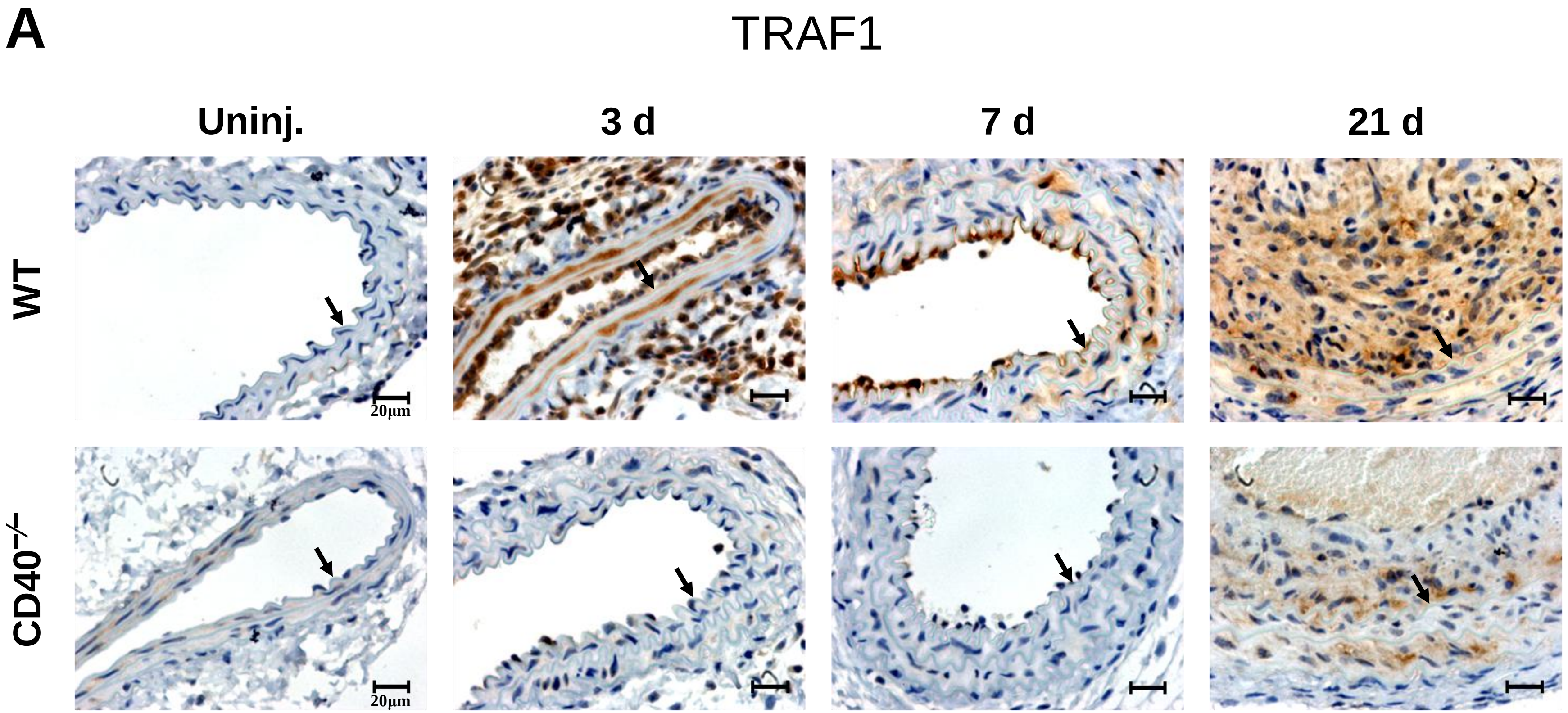

Fig. S2

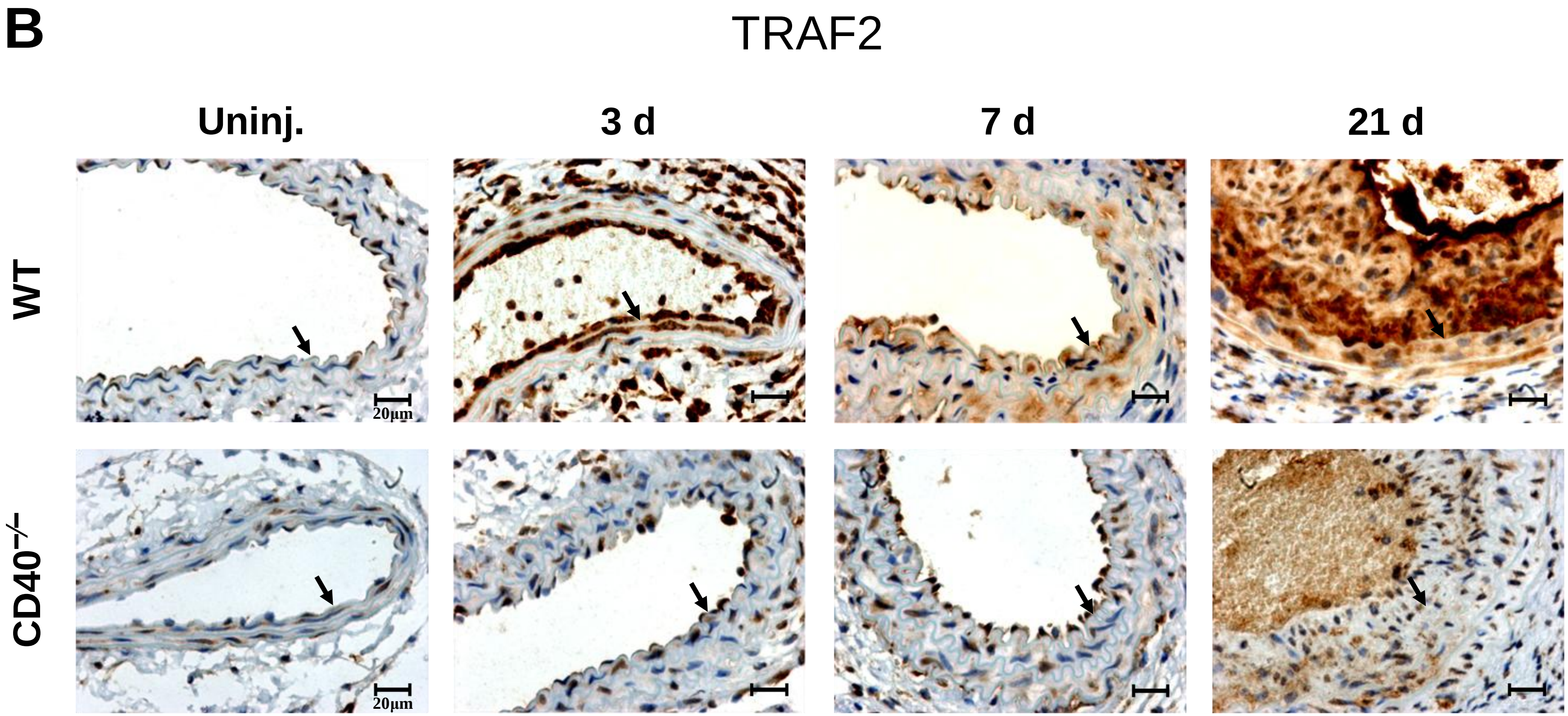

Fig. S2

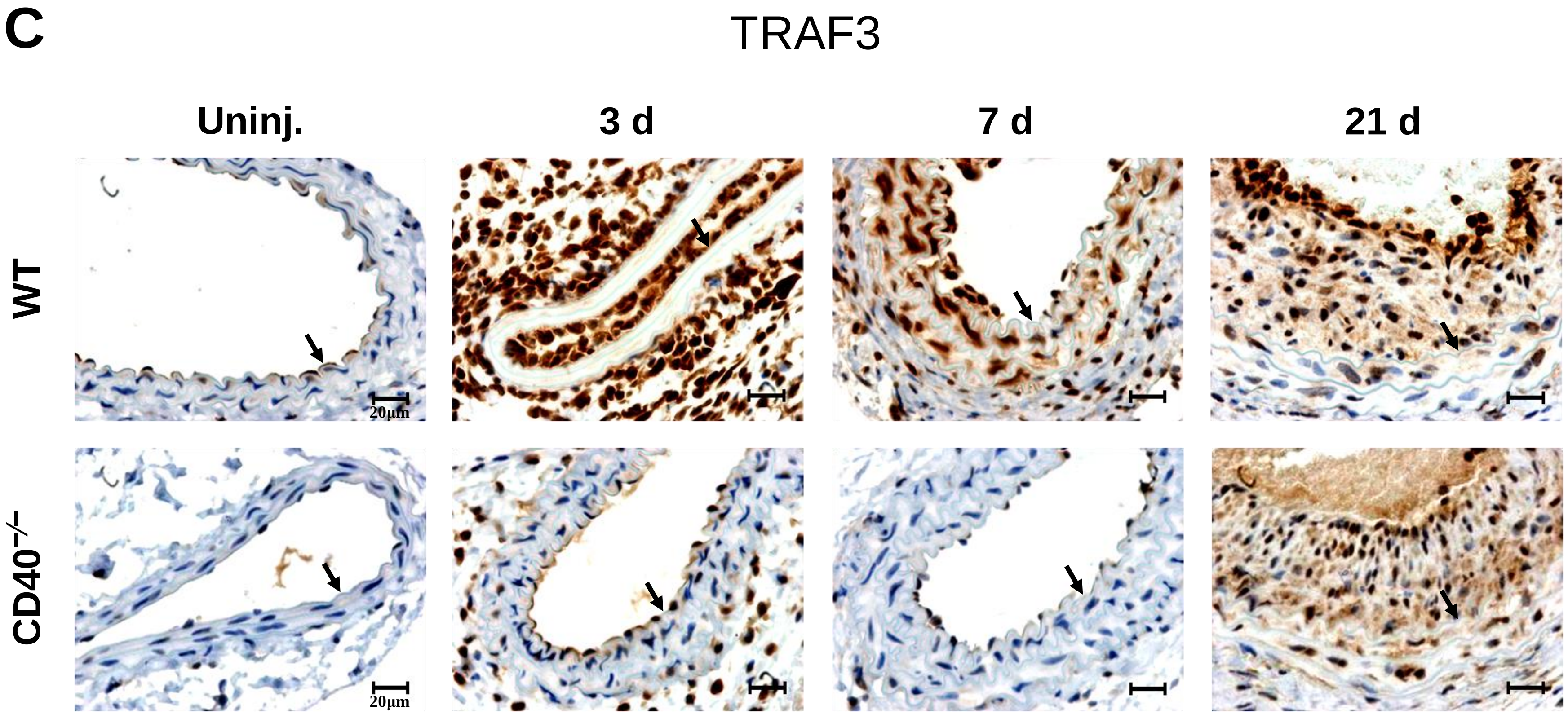

Fig. S2

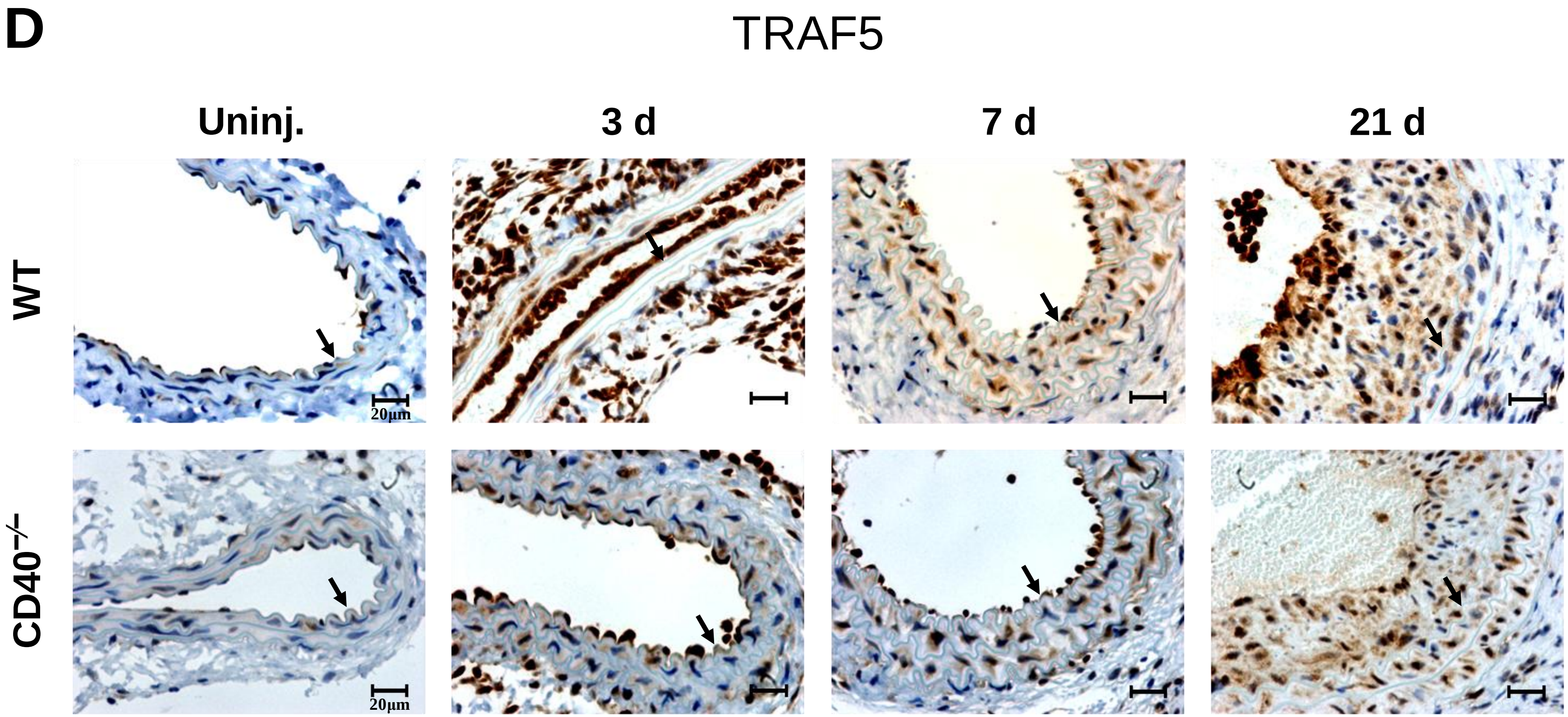

Fig. S2
