# Supplementary material for: CD40 Is Essential in the Upregulation of TRAF Proteins and NF-KappaB-Dependent Proinflammatory Gene Expression after Arterial Injury
Source: PLoS One. 2011 Aug 18;6(8):e23239. doi: 10.1371/journal.pone.0023239 (PMC3158063; doi:10.1371/journal.pone.0023239)
Supplement: Figure S3 — Decreased neointima formation and lumen stenosis after carotid artery ligation in CD40−/− mice. (A) Representative Elastic-stained sections (level 3) of carotid arteries 21d after injury in WT and CD40−/− mice (left panel). Schematic diagram of carotid artery ligation and tissue microtomy (right panel). Arrows indicate the internal elastic lamina. Scale bars: 50 µm. (B) Intima was measured at the 7 cross-section levels (120-µm intervals), and their mean intimal area was calculated. Intima/media ratio (C) and lumen stenosis ratio (D) at each level as well as their mean ratios were determined. n = 10 mice per group. Data are expressed as mean ± SEM. * P<0.05 and ** P<0.01 versus corresponding WT. (PDF) [file pone.0023239.s003.pdf]

Figure S3. ***Decreased neointimal formation and lumen stenosis after carotid artery ligation in CD40-/- mice***

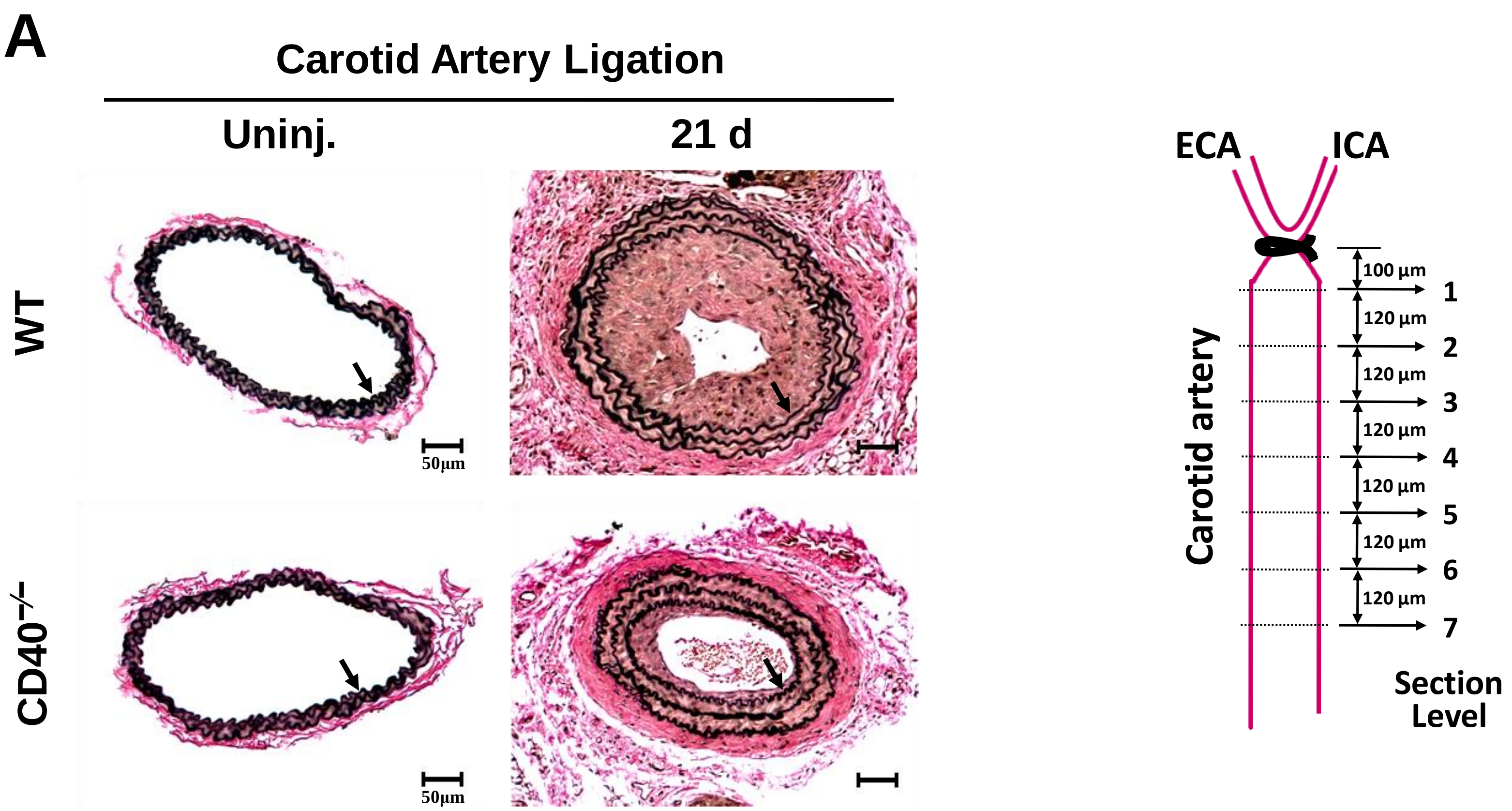
A
Carotid Artery Ligation
Uninj.
21 d
WT
CD40⁻/⁻
50μm
50μm
ECA
ICA
Carotid artery
100 μm
120 μm
1
2
3
4
5
6
7
Section Level

Fig. S3

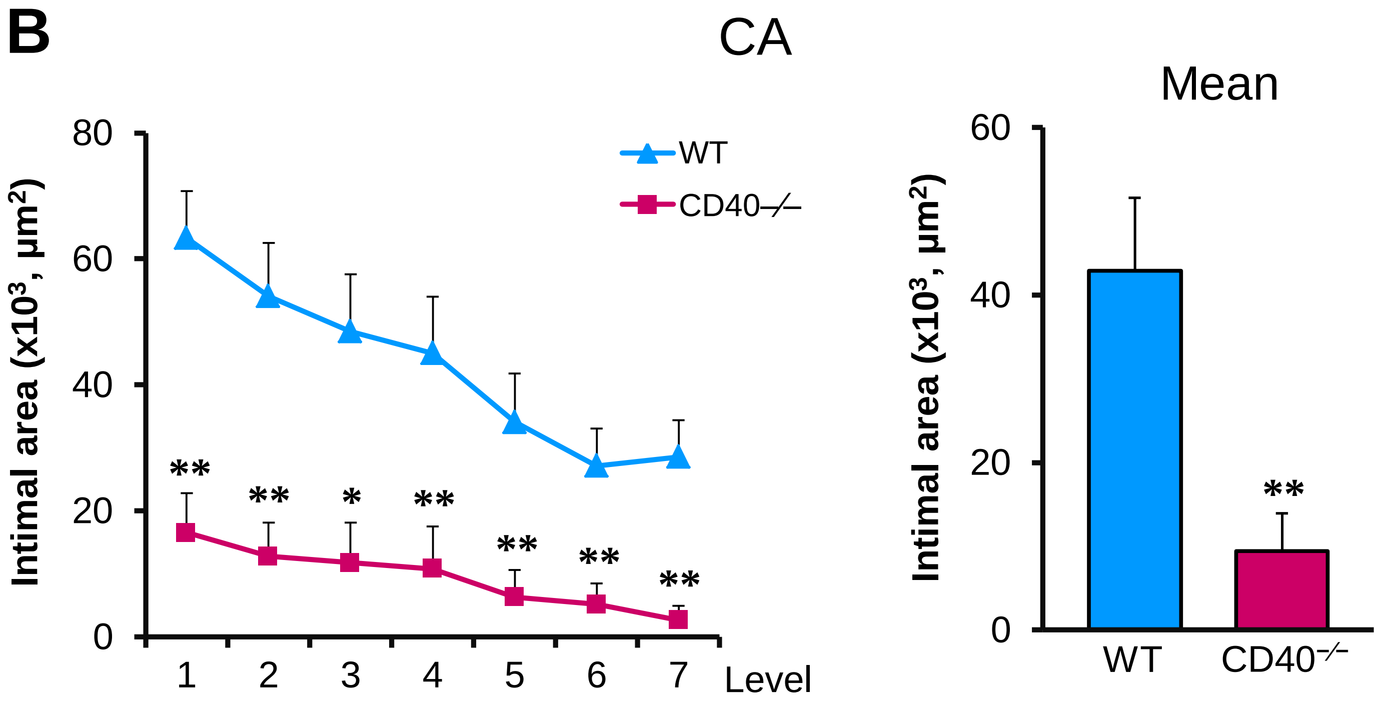
B
CA
Mean
80
60
40
20
0
Intimal area (x10³, µm²)
WT
CD40-/-
**
**
*
**
**
**
**
1
2
3
4
5
6
7
Level
60
40
20
0
Intimal area (x10³, µm²)
**
WT
CD40-/-

Fig.S3

C

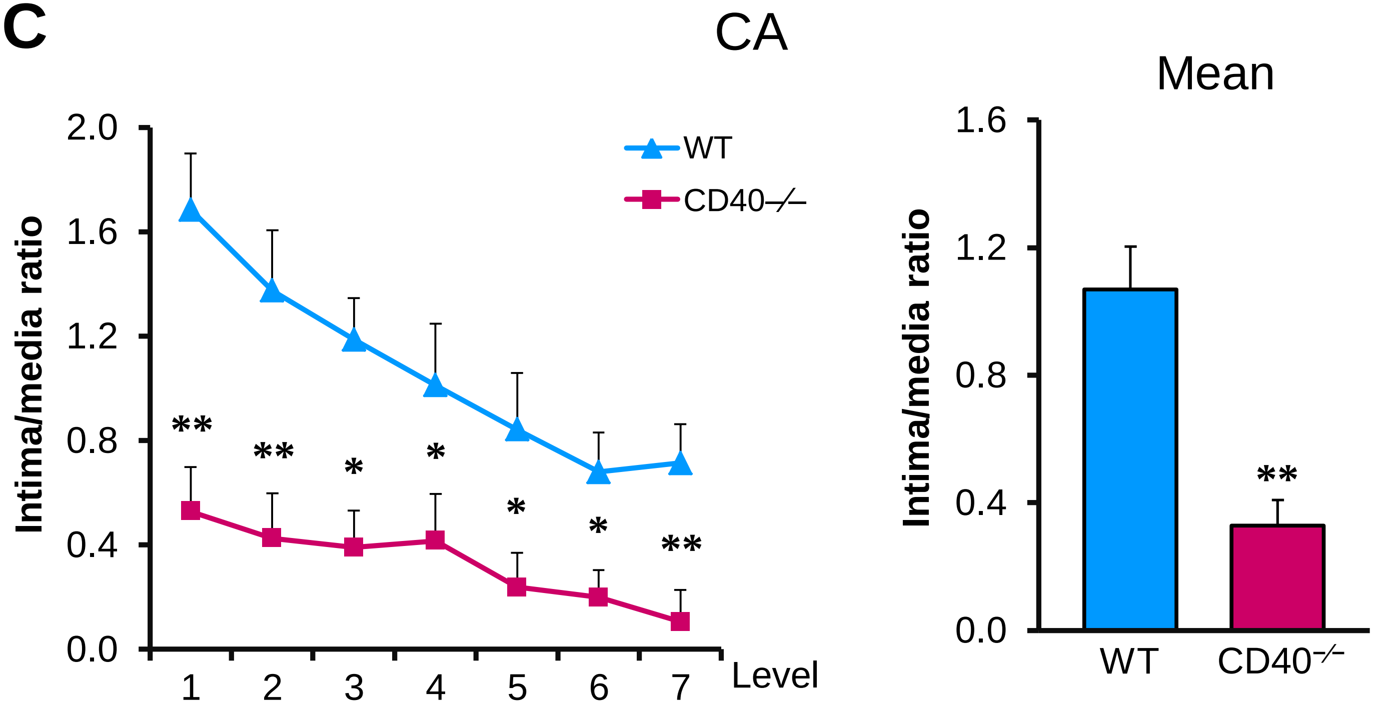
CA
Intima/media ratio
2.0
1.6
1.2
0.8
0.4
0.0
WT
CD40−/−
**
**
*
*
*
*
**
1
2
3
4
5
6
7
Level
Mean
Intima/media ratio
1.6
1.2
0.8
0.4
0.0
**
WT
CD40−/−

Fig. S3

D

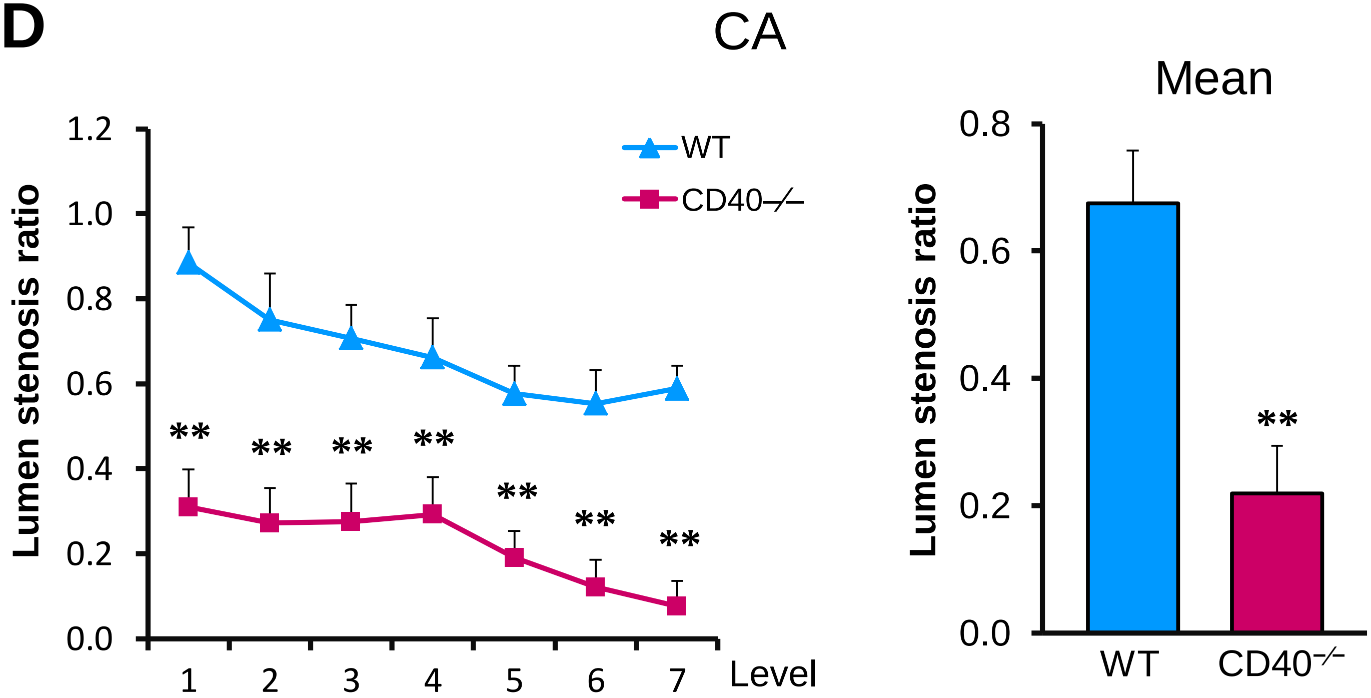
CA
Lumen stenosis ratio
1.2
1.0
0.8
0.6
0.4
0.2
0.0
WT
CD40−/−
**
1
2
3
4
5
6
7
Level
Mean
0.8
0.6
0.4
0.2
0.0
WT
CD40−/−

Fig. S3
