# Supplementary material for: CD40 Is Essential in the Upregulation of TRAF Proteins and NF-KappaB-Dependent Proinflammatory Gene Expression after Arterial Injury
Source: PLoS One. 2011 Aug 18;6(8):e23239. doi: 10.1371/journal.pone.0023239 (PMC3158063; doi:10.1371/journal.pone.0023239)
Supplement: Figure S4 — Effects of CD40 deficiency on arterial remodeling after injury. (A) Total vessel area was measured in carotid arteries and femoral arteries 21d after injury in WT and CD40−/− mice. n = 10 mice per group. Data are expressed as mean ± SEM. ** P<0.01 versus WT. Lumen area, media area, and total vessel area were measured in uninjured carotid arteries (B) and uninjured femoral arteries (C) from WT (n = 7 vessel) and CD40−/− mice (n = 6 vessel). Data are expressed as mean ± SEM. No differences in vessel geometry were found between WT and CD40−/− mice in uninjured arteries. (PDF) [file pone.0023239.s004.pdf]

Figure S4. **The impact of CD40 deficiency on vascular remodeling after vascular injury**

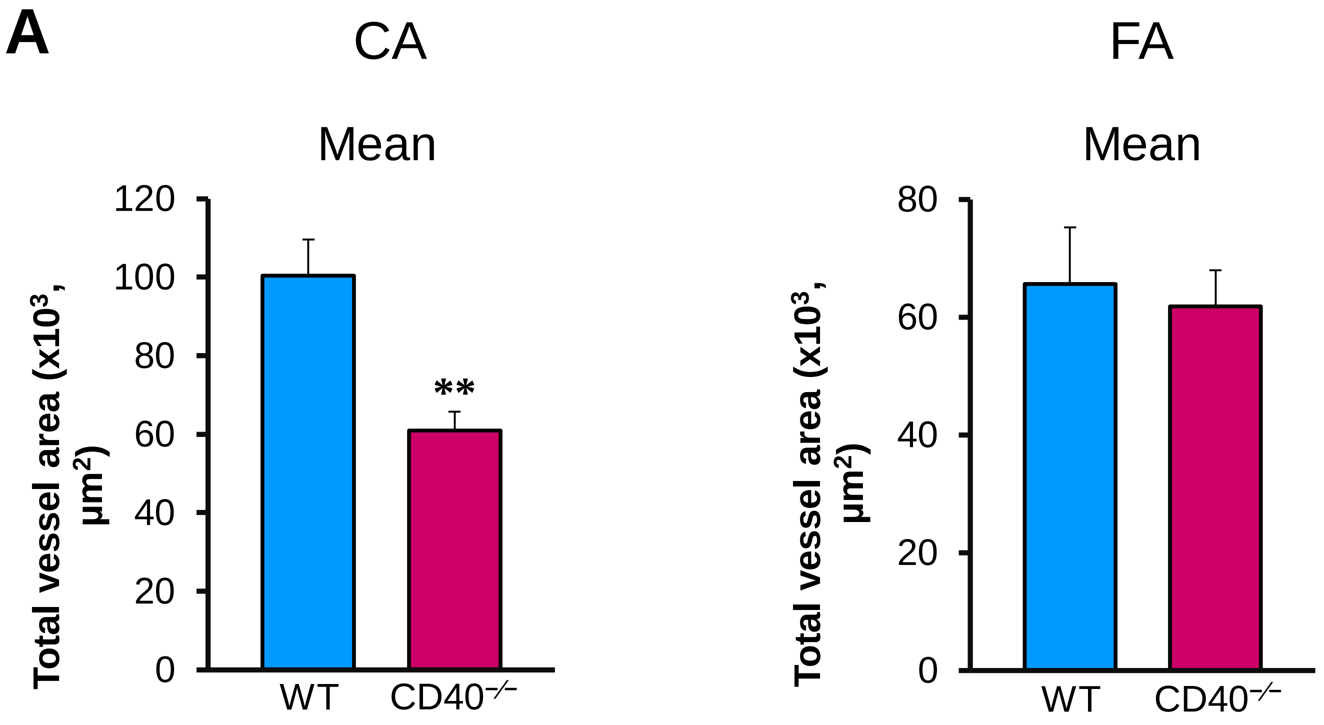
A
CA
Mean
120
100
80
60
40
20
0
Total vessel area (x10³, μm²)
**
WT
CD40⁻/⁻
FA
Mean
80
60
40
20
0
Total vessel area (x10³, μm²)
WT
CD40⁻/⁻

Fig. S4

B

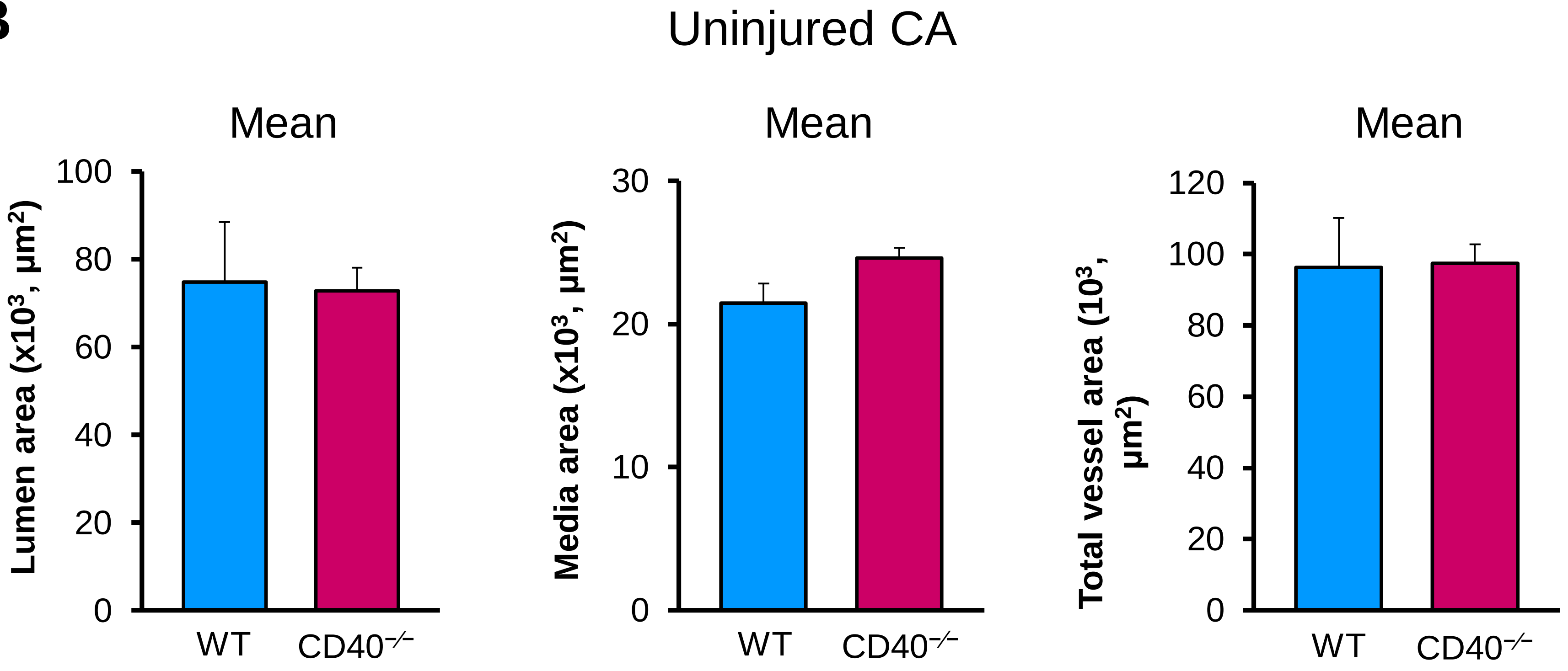

Uninjured CA
Mean
Mean
Mean
Lumen area (x10³, µm²)
Media area (x10³, µm²)
Total vessel area (10³, µm²)
100
80
60
40
20
0
30
20
10
0
120
100
80
60
40
20
0
WT
CD40−/−
WT
CD40−/−
WT
CD40−/−

Fig. S4

C

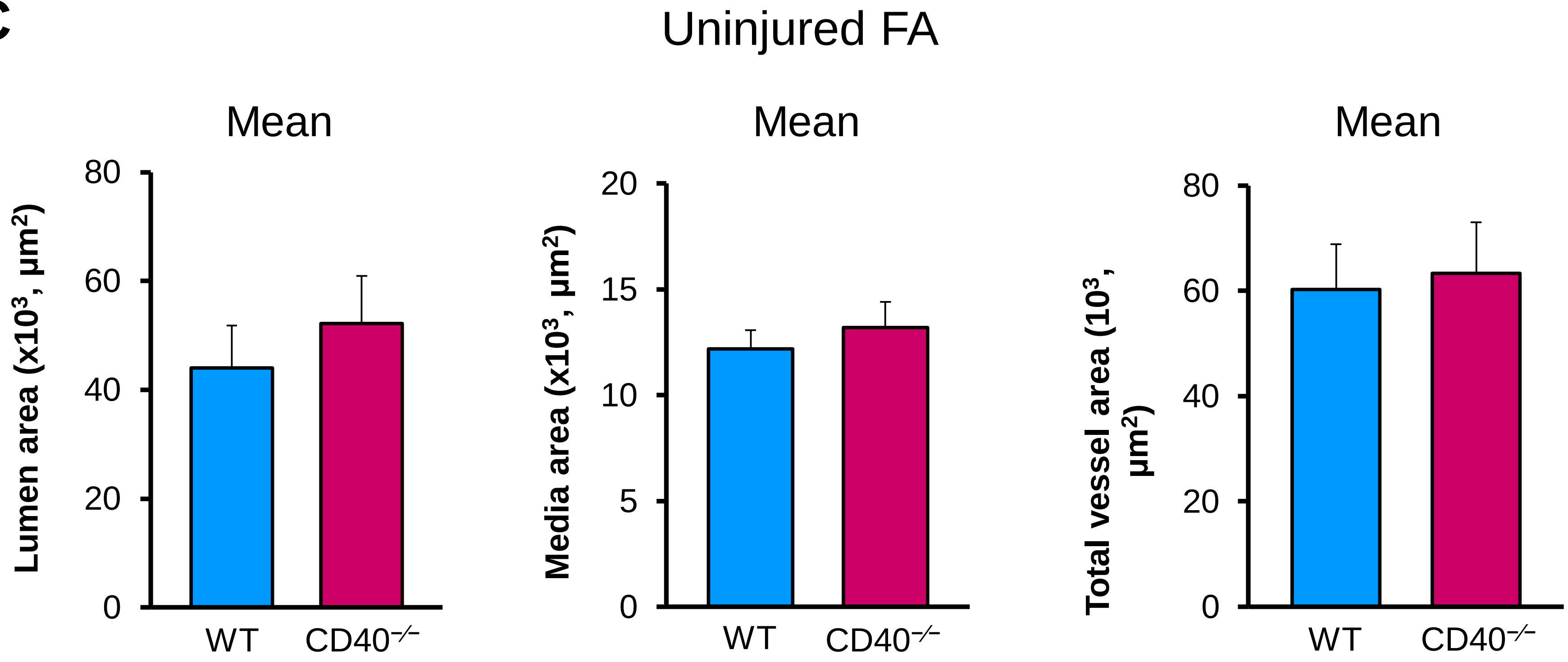

Uninjured FA
Mean
Lumen area (x10³, µm²)
80
60
40
20
0
WT
CD40−/−
Mean
Media area (x10³, µm²)
20
15
10
5
0
WT
CD40−/−
Mean
Total vessel area (10³, µm²)
80
60
40
20
0
WT
CD40−/−

Fig. S4
