# Supplementary figures and images for: CD40 Is Essential in the Upregulation of TRAF Proteins and NF-KappaB-Dependent Proinflammatory Gene Expression after Arterial Injury
Source: PLoS One. 2011 Aug 18;6(8):e23239. doi: 10.1371/journal.pone.0023239 (PMC3158063; doi:10.1371/journal.pone.0023239)

## Figure S5. Plasma MCP-1 and soluble ICAM-1 in CD40<sup>-/-</sup> mice and WT controls

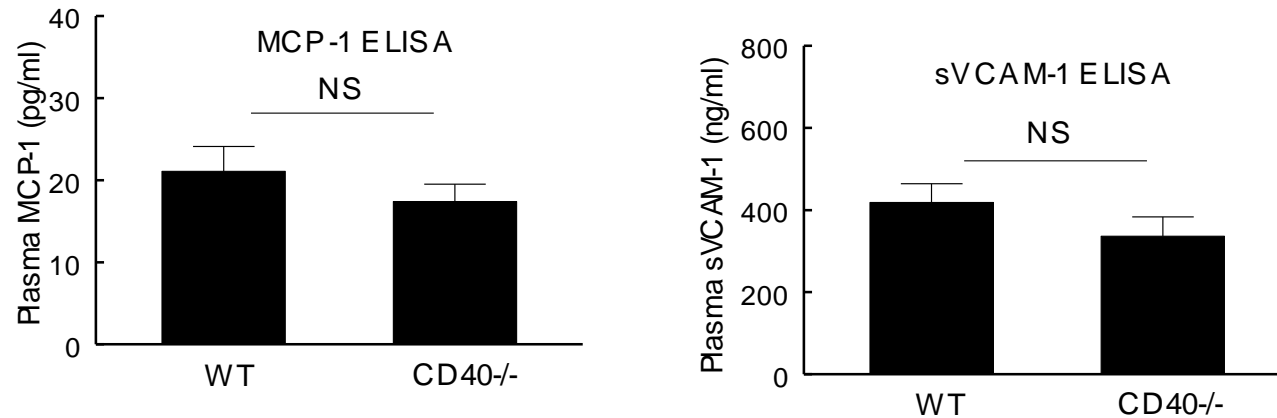

Fig. S5

Supplement: Figure S5 — Measurement of plasma levels of MCP-1 and soluble VCAM-1 using Quantikine Mouse ELISA Kits (R&D Systems) in CD40−/− mice and WT controls. NS: P>0.05. n = 5/group. (PDF) [file pone.0023239.s005.pdf]
