# Supplementary material for: CD40 Is Essential in the Upregulation of TRAF Proteins and NF-KappaB-Dependent Proinflammatory Gene Expression after Arterial Injury
Source: PLoS One. 2011 Aug 18;6(8):e23239. doi: 10.1371/journal.pone.0023239 (PMC3158063; doi:10.1371/journal.pone.0023239)
Supplement: Table S1 — Complete blood cell counts. (PDF) [file pone.0023239.s006.pdf]

## Table S1. Complete blood cell counts (1000/μL)

| Mice | White blood cell | Granulocytes | Lymphocytes | Monocytes | Platelets |
|---|---|---|---|---|---|
| C57Bl6 | 4.72±0.43 | 2.46±0.28 | 1.67±0.19 | 0.28±0.12 | 745±98 |
| CD40-/- | 4.65±0.45 | 2.37±0.26 | 1.63±0.18 | 0.27±0.13 | 821±101 |

Peripheral blood was collected from the retro-orbital plexus of mice anesthetized with isofluorane, using heparinized microhematocrit capillary tubes. Complete blood counts were performed using an automated veterinary hematology analyzer (VetABC, Scil Veterinary Diagnostics).
