# Supplementary material for: CD40 Is Essential in the Upregulation of TRAF Proteins and NF-KappaB-Dependent Proinflammatory Gene Expression after Arterial Injury
Source: PLoS One. 2011 Aug 18;6(8):e23239. doi: 10.1371/journal.pone.0023239 (PMC3158063; doi:10.1371/journal.pone.0023239)
Supplement: Table S2 — Primer sequences for real-time RT-PCR. (PDF) [file pone.0023239.s007.pdf]

Table S2. Primer sequences for quantitative RT-PCR

Primer	Primer sequence	PCR product size
**CD40**	F: 5'-CACTGATACCGTCTGTCATCCCT-3'<br>R: 5'-ATGACCAGCAGGGCTCGCAT-3'	[180 bp]
**ICAM-1**	F: 5'-CCTGCCTAAGGAAGACATGA-3'<br>R: 5'-CCCAGACTCTCACAGCATCT-3'	[222 bp]
**VCAM-1**	F: 5‘-CCCAAACAGAGGCAGAGTGT-3'<br>R: 5‘-CAGGATTTTGGGAGCTGGTA-3'	[150 bp]
**MCP-1**	F: 5'-GAAGGAATGGGTCCAGACAT-3'<br>R: 5'-ACGGGTCAACTTCACATTCA-3'	[127 bp]
**TNF-α**	F: 5’-CCCACTCTGACCCCTTTACT-3’<br>R: 5’-TTTGAGTCCTTGATGGTGGT-3’	[201 bp]
**IL-6**	F: 5’-CTACCCCAATTTCCAATGCT-3’<br>R: 5’-ACCACAGTGAGGAATGTCCA-3’	[187 bp]
**IL-1β**	F: 5’-CCCAACTGGTACATCAGCAC-3’<br>R: 5’-TCTGCTCATTCACGAAAAGG-3’	[180 bp]
**GAPDH**	F: 5’-CTGGAGAAACCTGCCAAGTA-3’<br>R: 5’-TGTTGCTGTAGCCGTATTCA-3’	[223 bp]
